# Behavioural and institutional drivers of green technology adoption: an extended technology acceptance model for sustainability transitions

**DOI:** 10.3389/fpsyg.2026.1723942

**Published:** 2026-03-13

**Authors:** Gonzalo Mariscal, Jesús Cáceres-Tello, José Javier Galán-Hernández

**Affiliations:** 1 Department of Computing and Technology, School of Architecture, Engineering, Science and Computing (STEAM), European University of Madrid, Madrid, Spain; 2 Department of Computer Systems and Computing, Faculty of Computer Science, Complutense University of Madrid, Madrid, Spain

**Keywords:** behavioural public policy, community support, green technology adoption, institutional trust, legitimacy, sustainability governance, technology acceptance model

## Abstract

The adoption of green technologies is a central component of sustainability transitions, yet it cannot be fully understood through individual cognitive evaluations alone. Increasing evidence suggests that citizens’ acceptance of environmentally oriented technologies is shaped by institutional and normative contexts that influence behavioural decision-making. In response to this challenge, this study develops a conceptual extension of the Technology Acceptance Model (TAM) to examine green technology acceptance from an integrative perspective grounded in environmental psychology and behavioural public policy. The analysis is based on a structured conceptual synthesis informed by bibliometric and semantic evidence from 789 Scopus-indexed articles published between 2021 and 2025, including early-indexed records from 2025 available at the time of data retrieval. Recurrent patterns within this corpus link technology acceptance research with institutional constructs such as trust, legitimacy, transparency, and community support. These insights are complemented by a structured expert elicitation exercise using the Analytic Hierarchy Process (AHP), applied in an exploratory manner to examine the relative salience of institutional dimensions within the proposed framework. Rather than offering empirical validation of behavioural relationships, the study advances a theoretically grounded conceptual model in which institutional trust and legitimacy operate as contextual conditions shaping the relationship between perceived usefulness, perceived ease of use, and behavioural intention in sustainability-related adoption contexts. Institutional transparency and community support further reinforce perceptions of fairness, credibility, and social endorsement. Overall, the resulting framework conceptualises green technology adoption as a socially and institutionally embedded process, providing a coherent analytical foundation for future empirical research and for the design of behavioural public policies aimed at supporting sustainability transitions.

## 1 Introduction

The ecological transition has moved beyond the realm of long-term aspiration and now constitutes an urgent and collective challenge. Climate change, biodiversity loss, and increasing pressures on urban systems are reshaping the conditions under which human wellbeing and environmental sustainability coexist. Addressing these challenges requires not only technological innovation but also sustained changes in individual and collective behaviour. Research in environmental psychology has consistently shown that how individuals perceive, conceptualise, and emotionally relate to nature conditions their willingness to engage in pro-environmental actions (Hatty et al., 2022).

Within this context, green technologies, understood as innovations designed to reduce environmental impacts through cleaner production processes, improved energy efficiency, and more sustainable consumption patterns, play a central role in achieving global sustainability objectives. International policy frameworks, such as the Sustainable Development Goals, explicitly recognise the diffusion of environmentally oriented technologies as a key lever for sustainability transitions (United Nations (UN), 2024). Yet, the societal uptake of these technologies cannot be explained solely by their technical performance or economic efficiency. Empirical evidence increasingly indicates that adoption processes are shaped by psychological, social, and institutional contexts that frame how individuals interpret technological change (Eppe et al., 2025).

Public policy has traditionally sought to promote technology adoption through regulatory instruments, economic incentives, and information-based interventions. While these approaches have generated measurable outcomes, they often rely on assumptions of homogeneous rationality and stable preferences that only partially capture real-world decision-making. From a policy design perspective, formulation processes unfold along a spectrum ranging from deliberate design to adaptive forms of non-design, which constrains the effectiveness of standardised and purely instrumental interventions (Howlett and Mukherjee, 2014). These limitations have contributed to growing interest in behavioural public policy, an approach that explicitly recognises the bounded, context-dependent, and socially embedded nature of human behaviour (Fleiß et al., 2024). Within this perspective, decision-making is understood as embedded within institutional environments characterised by trust, legitimacy, and normative expectations, which shape how citizens interpret public action and sustainability-oriented policies (Dryhurst et al., 2020).

The study of technology acceptance offers a valuable analytical entry point for understanding these dynamics. The Technology Acceptance Model (TAM), originally proposed by Davis (1989), has provided a robust framework for explaining adoption behaviour through two core constructs: perceived usefulness and perceived ease of use. Subsequent extensions, including the Unified Theory of Acceptance and Use of Technology (UTAUT), have broadened this perspective by incorporating social influence and facilitating conditions (Venkatesh et al., 2016). Meta-analytic evidence confirms the enduring relevance of TAM-based models across diverse application domains (Ibrahim et al., 2025). However, the original formulation of TAM remains primarily focused on individual cognitive evaluations and offers limited insight into the institutional and normative environments in which technology adoption takes place, particularly in governance-intensive and public-sector contexts (Mustofa et al., 2025).

From a normative and social-psychological perspective, legitimacy, social norms, and moral obligation have long been recognised as key drivers of collective behaviour. Classic work on normative conduct demonstrates that social norms function as powerful reference points guiding individual action within collective settings (Cialdini et al., 1991). In sustainability-related domains, ethical obligation, pro-environmental identity, and institutional transparency further condition technology-related decision-making by reinforcing perceptions of fairness, credibility, and procedural justice (Kumar et al., 2023; Alessandro et al., 2021). Together with public trust, these institutional dimensions shape citizens’ acceptance of both technological innovations and the policies through which they are promoted (Siegrist and Zingg, 2014).

**Figure 1:**
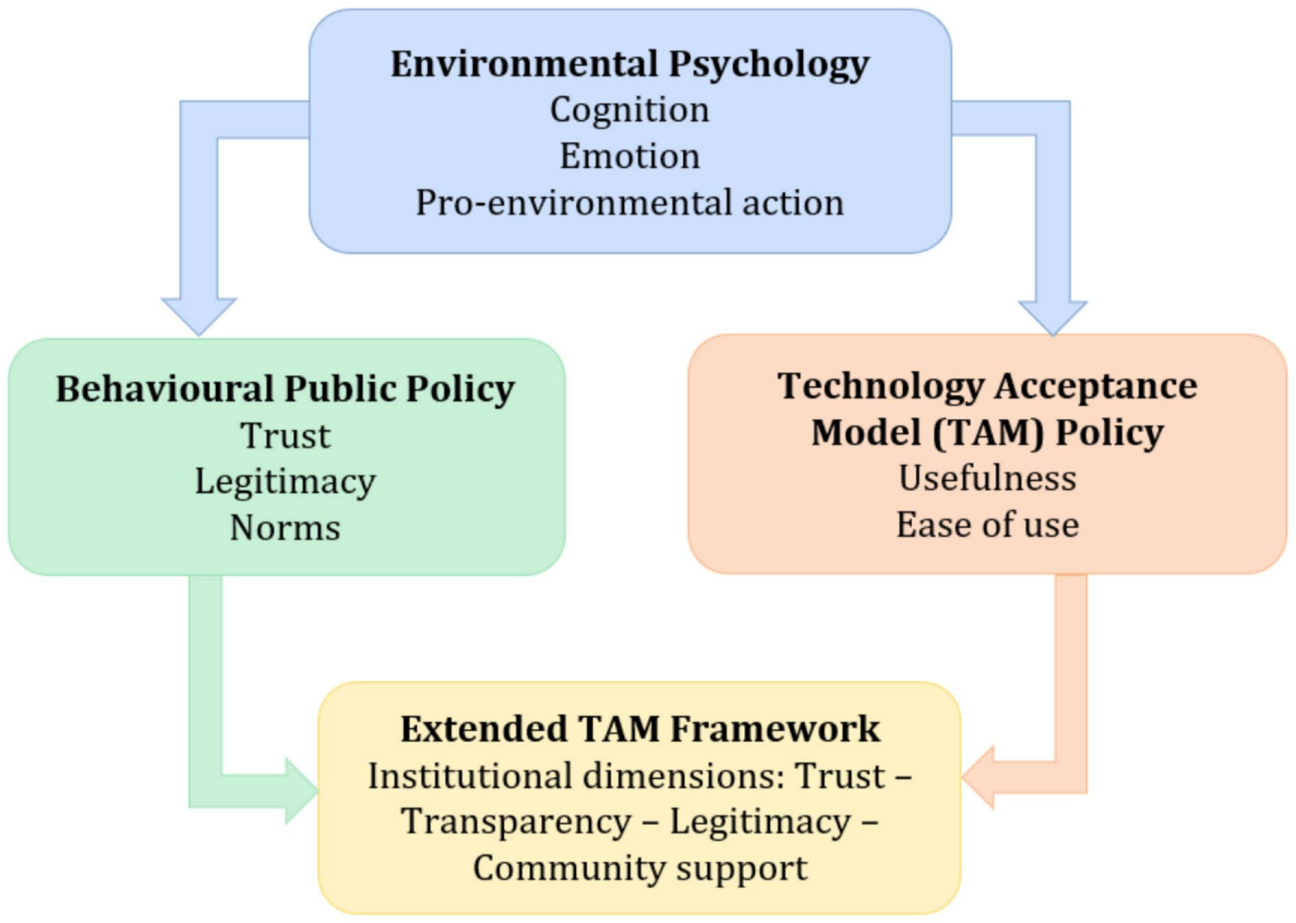
Conceptual positioning of theoretical domains. The figure does not imply empirically tested causal relationships. Source: authors’ elaboration.

Figure 1 situates the present study within this emerging interdisciplinary landscape by positioning environmental psychology, behavioural public policy, and technology acceptance research within a unified analytical perspective. The figure does not depict causal relationships or empirically tested effects but rather delineates the theoretical domains that converge in the analysis of green technology adoption.

Building on prior work examining behavioural and institutional determinants of technology adoption (Cáceres-Tello and Galán-Hernández, 2024), this study develops a conceptual extension of the Technology Acceptance Model tailored to sustainability contexts. Through a structured synthesis of bibliometric, semantic, and theoretical evidence, four institutional dimensions; trust, transparency, legitimacy, and community support, are identified as framing citizens’ acceptance of green technologies alongside the classical TAM constructs. From a theoretical standpoint, this research contributes to a more integrated understanding of sustainability transitions as processes shaped jointly by cognitive evaluations and institutional conditions. From an applied perspective, it provides a coherent conceptual basis for future empirical research and for the design of behavioural public policies that are sensitive to institutional context. The following section outlines the methodological strategy employed to develop this conceptual framework.

## 2 Materials and methods

Understanding the behavioural and institutional dimensions underlying the acceptance of green technologies requires a methodological approach that is both integrative and transparent. Rather than seeking to empirically test behavioural relationships or estimate causal effects, this study adopts a conceptual synthesis strategy aimed at structuring and consolidating dispersed theoretical insights. To this end, bibliometric evidence, semantic analysis, and structured expert elicitation are combined to develop an extended Technology Acceptance Model (TAM) adapted to sustainability-oriented and governance-intensive contexts.

**Figure 2:**
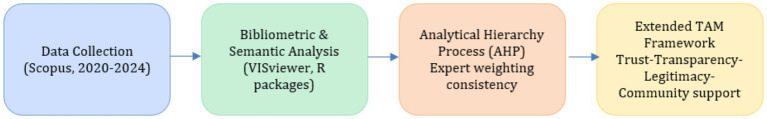
Methodological workflow of the study illustrating the four sequential stages: data collection (Scopus, 2020–2025), bibliometric and semantic analysis (VOSviewer, R), analytical hierarchy process (AHP) for expert weighting and consistency validation, and conceptual integration into the extended technology acceptance model (TAM) framework. Source: authors’ elaboration.

The methodological process was organised into four sequential stages: (1) systematic collection of relevant scientific literature indexed in Scopus; (2) bibliometric and semantic analysis of the selected corpus; (3) structured expert elicitation using the Analytic Hierarchy Process (AHP) to organise institutional dimensions; and (4) conceptual integration of behavioural and institutional constructs into the extended TAM framework. This sequence reflects a progressive analytical logic in which descriptive mapping and expert-based structuring jointly support the development of the proposed conceptual model, as outlined in Figure 2.

The following subsections describe each phase of the methodological process in detail, outlining the research design, data collection strategy, analytical procedures, and conceptual integration adopted in the study.

### 2.1 Research design

The study followed a conceptual mixed-methods design aimed at integrating behavioural, psychological, and institutional perspectives into a unified analytical framework. Instead of testing predefined hypotheses or estimating statistical relationships, the research focused on organising, comparing, and synthesising theoretical constructs relevant to citizens’ acceptance of green technologies. This approach is grounded in the principles of systematic conceptual synthesis, which emphasise the structured integration of heterogeneous bodies of knowledge while maintaining theoretical coherence and methodological transparency (Tranfield et al., 2003).

The methodological logic combines bibliometric mapping, expert reasoning, and hierarchical structuring of concepts to generate analytical depth without relying on inferential statistical testing. As outlined in the overall workflow, the research unfolds through four interrelated phases, data collection, bibliometric and semantic analysis, analytical hierarchy structuring, and conceptual integration, each contributing complementary insights that progressively refine the extended TAM framework linking environmental psychology, behavioural public policy, and technology acceptance theory.

### 2.2 Data collection (Scopus 2021–2025)

The bibliographic corpus was compiled using the Scopus database, selected for its broad multidisciplinary coverage and the consistency of its metadata in bibliometric research on technology acceptance and sustainability (Rosli et al., 2022). Scopus is widely used in reviews addressing behavioural, environmental, and policy-oriented research, making it suitable for mapping conceptual developments in this domain.

The literature search targeted publications referring to the Technology Acceptance Model (TAM) or closely related constructs, including technology adoption, intention to use, and psychological or social determinants of technology-related behaviour. The search strategy was implemented through a structured Boolean query combining technological, psychological, social, and environmental domains. The full search query, including all operators and field restrictions, is provided in Supplementary Text 1. The query was restricted to peer-reviewed journal articles published in English between 2021 and 2025. Publications dated 2025 correspond to early-indexed records available at the time of data retrieval (October 2025), ensuring a temporally coherent and comprehensive dataset.

After applying subject-area filters covering Psychology, Social Sciences, Environmental Science, Business, Management and Accounting, Decision Sciences, and Computer Science, the final corpus comprised 789 peer-reviewed journal articles. Bibliographic metadata, including authors, titles, abstracts, keywords, affiliations, sources, citations, and DOIs, were exported for subsequent bibliometric and semantic analysis. Data preprocessing and structuring followed established mapping procedures described by Van Eck and Waltman (2010). To preserve conceptual alignment with the proposed framework, the dataset included journals specialising in environmental psychology, behavioural science, sustainability, and technology acceptance, ensuring consistency between the empirical corpus and the theoretical domains informing the extended TAM.

### 2.3 Bibliometric and semantic analysis

Bibliometric and semantic analyses were conducted to identify conceptual patterns and relational structures within the selected corpus. The objective of this phase was to explore how institutional constructs—such as trust, transparency, legitimacy, and community support—are positioned within the broader literature on green technology acceptance and behavioural public policy.

Bibliometric mapping focused on author keyword co-occurrence networks to capture dominant themes and their interconnections. Keywords were harmonised by merging lexical variants (for example, “green technology” and “sustainable technology”) and by removing generic or non-informative terms. A minimum co-occurrence threshold was applied to balance conceptual inclusiveness with analytical clarity. Semantic analysis complemented this mapping by examining contextual relationships among key terms across titles, abstracts, and keywords, capturing not only frequency but also semantic proximity within academic discourse. Text preprocessing procedures included lemmatisation, stop-word removal, and the standardisation of spelling variants across British and American English.

The conceptual structure derived from this combined analysis provides the analytical foundation for the extended Technology Acceptance Model. The thematic clusters emerging from the bibliometric mapping are presented and discussed in Section 3.2, where they are interpreted in relation to the behavioural, environmental, and institutional dimensions of green technology acceptance.

### 2.4 Analytical hierarchy and model integration

This phase establishes a structured link between the conceptual synthesis developed in the previous stages and the organisation of its key dimensions. Rather than pursuing quantitative operationalisation or empirical validation, the analytical framework serves to systematically organise and compare behavioural and institutional constructs identified in the literature.

To support this structuring process, the study employed the Analytic Hierarchy Process (AHP) as a method of structured expert elicitation. AHP is a multi-criteria decision-making approach that enables transparent pairwise comparisons among conceptual dimensions, allowing expert judgement to be articulated in a consistent and reproducible manner (Tavana et al., 2023). Its applicability for organising qualitative assessments in complex environmental and sustainability-related contexts has been documented in recent studies (Ozgur, 2024).

Within this framework, four institutional dimensions; trust, transparency, legitimacy, and community support, were conceptually compared with the two core constructs of the original Technology Acceptance Model: perceived usefulness and perceived ease of use. These comparisons were intended to explore the relative positioning of institutional and behavioural dimensions within the proposed framework, rather than to estimate causal effects or behavioural parameters. The internal coherence of expert judgements was assessed using the consistency ratio criterion (CR < 0.1), ensuring that the resulting comparative structure reflected a reasoned synthesis of expert perspectives rather than arbitrary prioritisation.

### 2.5 Conceptual integration: extended TAM framework

The conceptual integration phase synthesises the behavioural and institutional dimensions identified in the preceding analyses into a unified theoretical framework. This stage connects the literature synthesis, the bibliometric mapping, and the expert-based structuring process, leading to the formulation of an extended Technology Acceptance Model (TAM) adapted to sustainability- and policy-oriented contexts.

The proposed framework retains the two foundational constructs of the original TAM, perceived usefulness and perceived ease of use, while incorporating four complementary institutional dimensions: trust, transparency, legitimacy, and community support. These dimensions represent institutional conditions that shape how individuals interpret, evaluate, and respond to sustainable technological innovations, particularly in governance-intensive settings where adoption decisions are closely intertwined with public authority, collective norms, and policy credibility.

**Figure 3:**
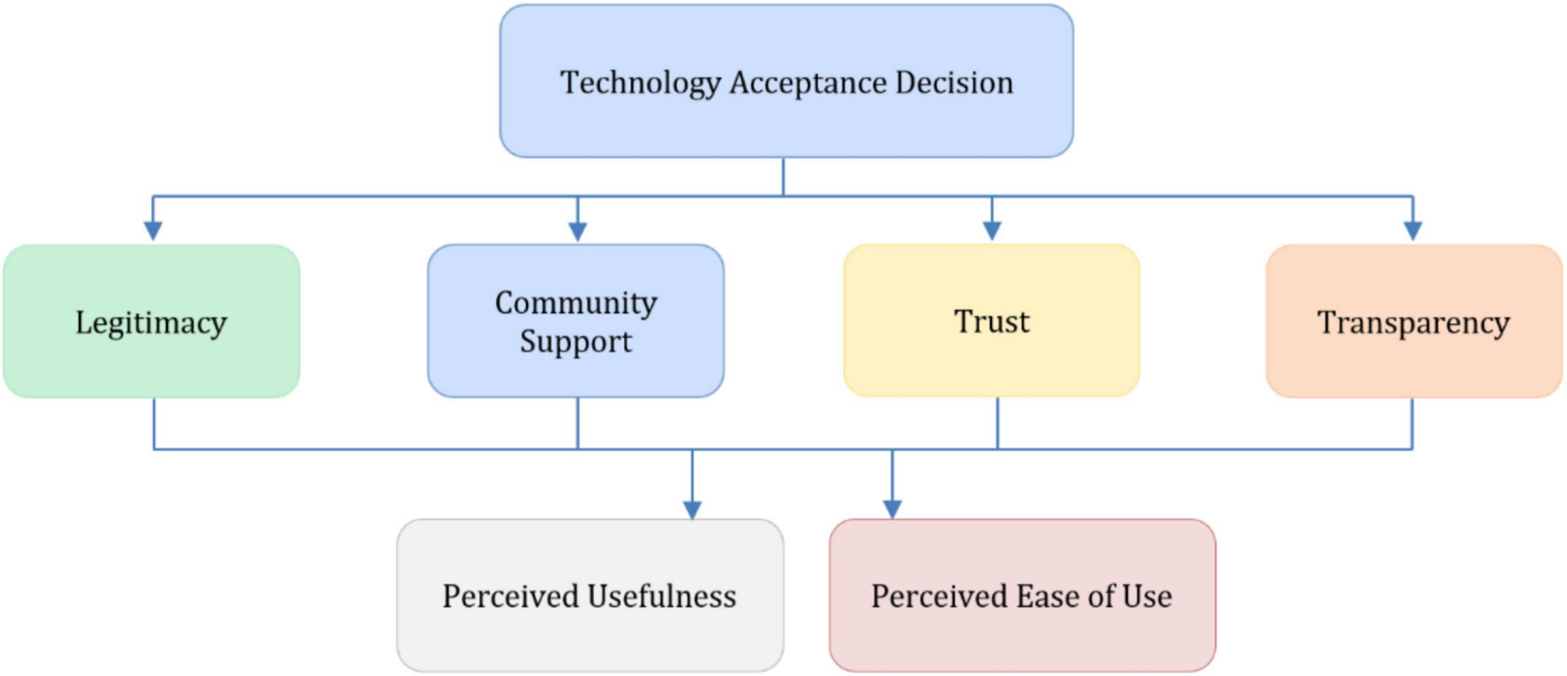
Extended Technology Acceptance Model (TAM) integrating behavioural (perceived usefulness, perceived ease of use) and institutional (trust, transparency, legitimacy, community support) dimensions. The figure represents a conceptual articulation of individual cognitive evaluations and institutional conditions framing technology acceptance in environmental and social contexts. Source: authors’ elaboration (based on Scopus data, retrieved October 2025).

The structure of the extended model is illustrated in Figure 3, which presents the conceptual positioning of behavioural and institutional elements within the proposed framework. The figure does not depict empirically tested causal relationships but rather provides a structured representation of how individual cognitive evaluations and institutional conditions are conceptually connected in the context of green technology acceptance.

By integrating these dimensions, the model captures the interaction between individual-level cognitive assessments and collectively mediated institutional signals. Institutional variables do not replace behavioural determinants but condition the circumstances under which perceived usefulness and perceived ease of use translate into behavioural intention. Trust and legitimacy shape expectations regarding fairness and reliability, transparency influences perceived accountability, and community support reflects social endorsement. Together, these dimensions frame technology acceptance as a socially embedded process rather than an isolated cognitive choice.

The extended TAM developed in this section provides the conceptual foundation for the analytical discussion presented in the following sections, supporting the interpretation of the bibliometric patterns and expert-based structuring results.

### 2.6 Descriptive overview of the literature corpus

The final corpus comprised 789 peer-reviewed journal articles published between 2021 and 2025, reflecting the growing academic interest in the intersection of behavioural, technological, and environmental perspectives on green technology adoption. Publications indexed in 2025 correspond to early-access records available at the time of data retrieval and are therefore interpreted descriptively. Across the period analysed, the annual volume of publications shows a clear upward tendency, increasing from 88 articles in 2021 to 276 in 2025, suggesting a progressive consolidation of behavioural and institutional approaches within sustainability- and technology-oriented research.

In terms of publication outlets, a relatively small group of journals accounted for a substantial share of the corpus. Sustainability (Switzerland) emerged as the most prominent source with 80 articles, followed by Education and Information Technologies (18), International Journal of Environmental Research and Public Health (17), International Journal of Human–Computer Interaction (13), and Technology in Society (11). This distribution illustrates the multidisciplinary character of the field, spanning environmental sciences, information systems, education, and behavioural research.

The main descriptive characteristics of the corpus are summarised in Table 1, which reports temporal coverage, citation indicators, and the most recurrent author keywords extracted from the Scopus dataset.

**Table 1:** Descriptive characteristics of the literature corpus (2021–2025).

Indicator	Description	Value/range
Period covered	Years of publication	2021–2025
Total documents analysed	Number of journal articles retrieved	789
Annual distribution	2021 (88); 2022 (121); 2023 (119); 2024 (185); 2025 (276)	–
Most frequent journals	*Sustainability (Switzerland)* (80); *Education and Information Technologies* (18); *International Journal of Environmental Research and Public Health* (17); *International Journal of Human–Computer Interaction* (13); *Technology in Society* (11)	–
Mean citations per article	–	14.4
Citation range	Minimum–maximum citations	0–540
Most recurrent author keywords	*technology acceptance model* (88); *technology acceptance* (73); *UTAUT* (50); *sustainability* (43); *UTAUT2* (36); *technology adoption* (33); *higher education* (31); *artificial intelligence* (30); *behavioural intention* (29); *structural equation modelling* (24)	–
Language	Articles written in English	100%

Source: authors’ elaboration based on Scopus data (retrieved October 2025).

Keyword frequencies further underscore the central position of the Technology Acceptance Model within the corpus, alongside closely related constructs such as technology acceptance, UTAUT, and sustainability. The recurrence of application-oriented terms, including artificial intelligence, higher education, and behavioural intention, points to an increasing diversification of empirical domains and analytical perspectives in which technology acceptance frameworks are being applied. Additional descriptive patterns are provided in Supplementary Figures S1–S4.

## 3 Conceptual and analytical finding

This section presents the conceptual and analytical findings derived from the bibliometric mapping and structured expert elicitation procedures outlined in the previous section. Rather than reporting empirical results or statistically validated relationships, the analysis focuses on identifying recurrent thematic configurations, conceptual associations, and expert-based analytical emphases that characterise the contemporary literature on green technology adoption. The findings are organised following a progressive analytical logic, combining keyword co-occurrence analysis and structured expert elicitation to provide a conceptually grounded basis for interpreting the extended Technology Acceptance Model (TAM) proposed in this study.

The analyses presented in this section are interpreted descriptively and conceptually. Co-occurrence structures and expert-based weightings are not treated as evidence of causal effects or predictive relationships, but as indicators of conceptual convergence and analytical orientation within the literature. This perspective allows the findings to inform theory development and framework consolidation while remaining consistent with the exploratory and theory-building objectives of the study.

### 3.1 Keyword co-occurrence and thematic structure

The keyword co-occurrence analysis offers a descriptive overview of the main thematic patterns emerging from the 789 articles included in the literature corpus. Using VOSviewer (version 1.6.20), a co-occurrence network was generated based on author-provided keywords, applying a minimum occurrence threshold to ensure conceptual clarity while retaining the diversity of perspectives represented in the field. Each node in the network corresponds to an author keyword, with node size indicating relative frequency and spatial proximity reflecting the strength of co-occurrence relationships.

**Figure 4:**
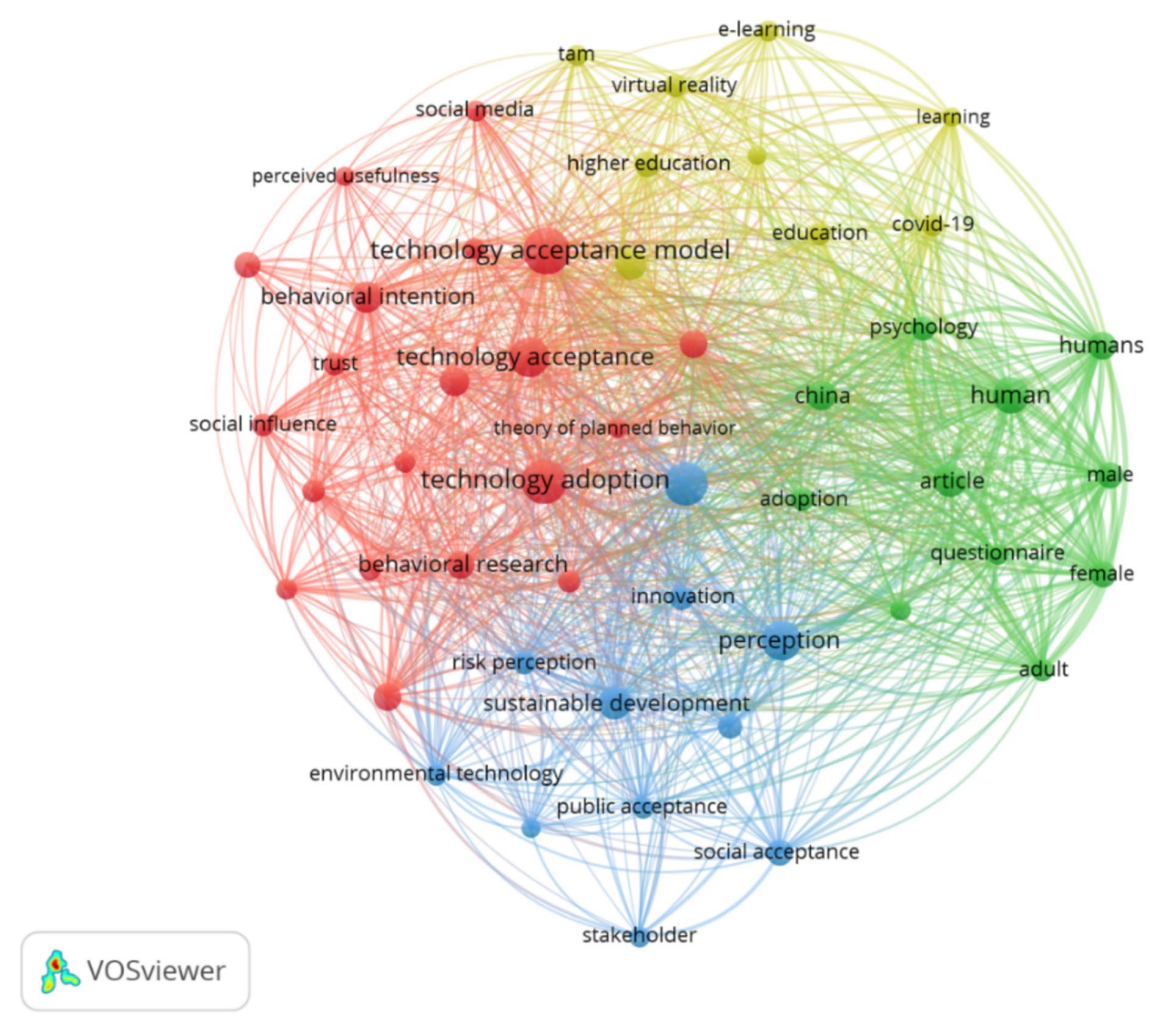
Co-occurrence network of author keywords (minimum 20 occurrences) generated using VOSviewer 1.6.20. Four color-coded clusters represent the conceptual structure of the field: behavioural and technological adoption (red), sustainability and environmental perception (green), psychosocial and demographic factors (blue), and educational applications (yellow). Source: authors’ elaboration based on Scopus data (n = 789, retrieved October 2025).

The resulting network, illustrated in Figure 4, reveals a structured configuration of interconnected thematic areas rather than isolated research streams. Four color-coded clusters can be distinguished, each representing a dominant orientation within the contemporary literature on green technology adoption.

The first cluster (red) centres on behavioural and technological adoption frameworks, with a strong emphasis on the Technology Acceptance Model (TAM), UTAUT, behavioural intention, and processes of digital transformation. This cluster reflects the longstanding analytical focus on individual-level cognitive evaluations within technology acceptance research, consistent with cumulative evidence from TAM-based meta-analytical syntheses (Ibrahim et al., 2025). Recent contributions within this cluster also highlight the psychosocial implications of emerging digital technologies, particularly artificial intelligence, in educational contexts, underlining the relevance of acceptance models that extend beyond instrumental performance (Jardón Gallegos et al., 2024).

The second cluster (green) is oriented towards sustainability and environmental perception, encompassing keywords related to green technology, environmental behaviour, sustainable development, and climate change. This grouping highlights the increasing integration of environmental objectives into technology adoption studies, situating acceptance processes within broader sustainability and policy discourses. The third cluster (blue) brings together psychosocial and institutional dimensions, including trust, attitudes, perception, legitimacy, and institutional quality, signalling a growing analytical emphasis on the institutional and normative contexts in which technology adoption decisions are embedded. The fourth cluster (yellow) relates to educational and applied contexts, with a concentration of keywords associated with higher education, e-learning, artificial intelligence, and digital literacy.

Taken together, the co-occurrence structure delineates a multidimensional conceptual landscape in which behavioural, environmental, and institutional perspectives intersect. Rather than indicating causal relationships, the observed clustering patterns point to areas of thematic convergence and analytical emphasis within the literature. In particular, the recurrent proximity between cognitive constructs (such as perceived usefulness and perceived ease of use) and institutional concepts (such as trust and legitimacy) signals an ongoing shift towards more integrative approaches to understanding sustainable technology adoption. These thematic patterns informed the subsequent use of structured expert elicitation to further organise and interpret key behavioural and institutional dimensions within the extended TAM framework.

### 3.2 Structured expert perspectives from the analytic hierarchy process

The Analytic Hierarchy Process (AHP) was employed as a structured expert elicitation tool to organise and comparatively assess the positioning of key behavioural and institutional dimensions identified through the bibliometric and semantic analyses. Rather than serving as a method for empirical validation or parameter estimation, AHP was used to facilitate transparent and systematic articulation of expert perspectives in a complex, multi-dimensional conceptual setting (Tavana et al., 2023).

**Figure 5:**
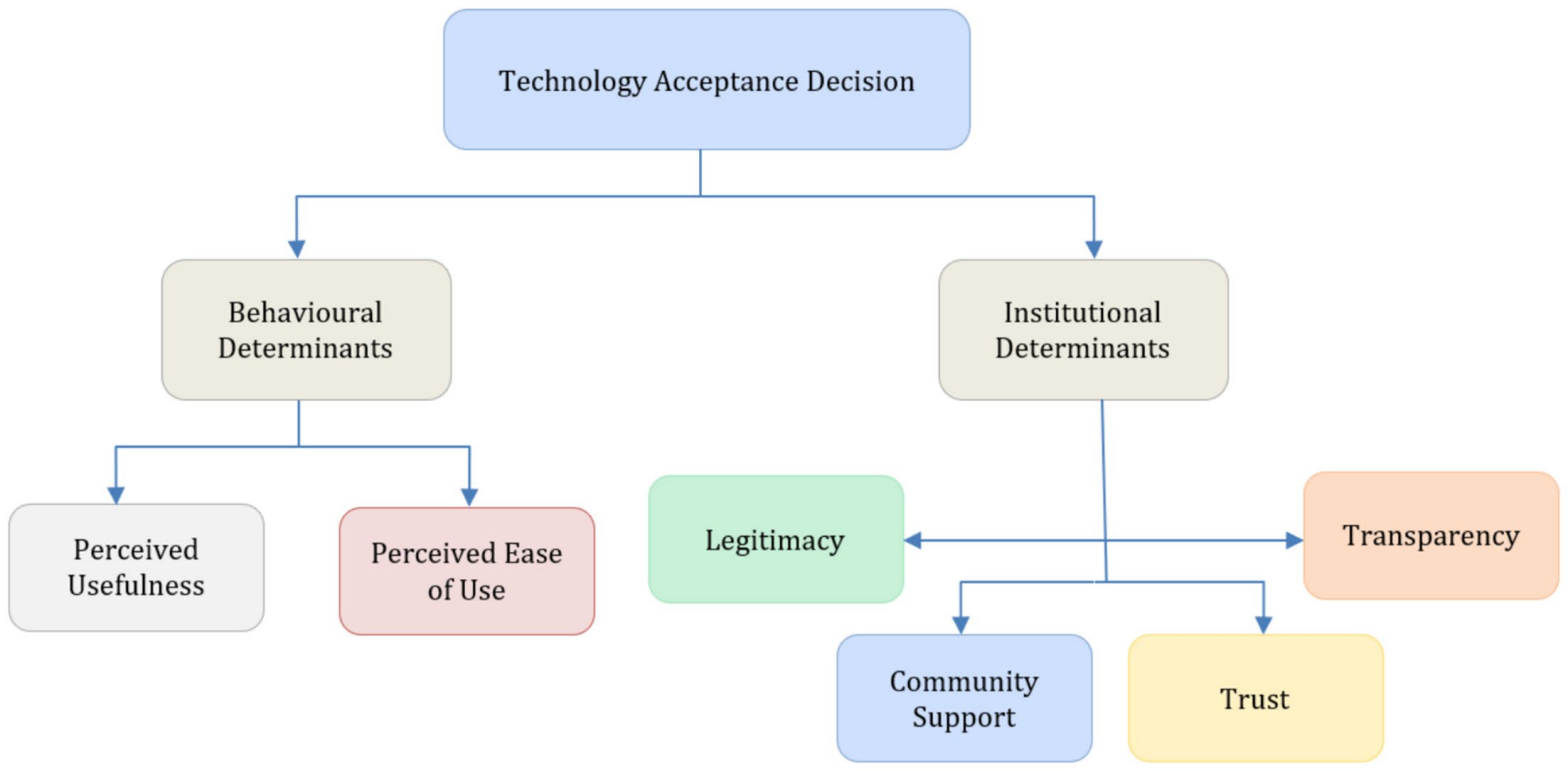
Analytical Hierarchy Process (AHP) structure integrating behavioural and institutional determinants within the extended Technology Acceptance Model (TAM). The model depicts the hierarchical relationships between the overall goal (technology acceptance decision), higher-level constructs (behavioural and institutional determinants), and mediating variables (legitimacy, trust, community support, transparency). Source: authors’ elaboration based on Scopus data and expert evaluation (retrieved October 2025).

As illustrated in Figure 5, the analytical structure was organised into three hierarchical levels. The upper level represents the overarching analytical focus on citizens’ acceptance of green technologies. The second level distinguishes between two broad dimensions derived from the extended Technology Acceptance Model (TAM): behavioural determinants, represented by perceived usefulness and perceived ease of use, and institutional determinants, encompassing trust, transparency, legitimacy, and community support. The third level comprises the specific constructs considered within each dimension, as evaluated by a panel of six experts with backgrounds in environmental psychology, governance, and behavioural public policy.

This hierarchical configuration does not seek to quantify behavioural effects or establish causal relationships. Instead, it provides an analytical representation of how experts conceptually differentiate, relate, and prioritise behavioural and institutional considerations when reflecting on sustainable technology adoption. Expert judgements were elicited through pairwise comparisons using the standard AHP scale, and internal coherence was assessed using the consistency ratio criterion (CR < 0.1), ensuring acceptable logical consistency across expert evaluations.

The resulting comparative patterns are summarised in Table 2, which reports the normalised weights assigned to each institutional construct within the proposed framework.

**Table 2:** Normalised AHP weights of the mediating variables in the extended TAM framework.

Construct	Category	Normalised weight	Consistency ratio (CR)
Trust	Institutional	0.34	<0.1
Legitimacy	Institutional	0.28	<0.1
Transparency	Institutional	0.22	<0.1
Community support	Institutional	0.16	<0.1

Source: authors’ elaboration based on expert evaluations (*n* = 6).

The distribution of weights indicates that experts consistently attributed greater analytical prominence to trust and legitimacy, followed by transparency and community support. These values should not be interpreted as empirically validated effect sizes or predictive parameters. Rather, they reflect how experts conceptually prioritise institutional dimensions when considering the conditions under which behavioural acceptance of green technologies is likely to occur. Taken together, these structured expert perspectives reinforce the conceptual relevance of institutional conditions and contribute to the consolidation of the extended TAM framework without implying statistical validation or behavioural prediction.

### 3.3 Conceptual consolidation of the extended technology acceptance model

Building on the thematic regularities identified through bibliometric mapping and the structured perspectives elicited via the Analytic Hierarchy Process, this section consolidates the extended Technology Acceptance Model (TAM) developed in this study. The proposed framework integrates behavioural, institutional, and normative dimensions into a single conceptual structure designed to reflect the complexity of green technology adoption in sustainability-oriented contexts.

Rather than expanding the model through additional explanatory variables or engaging in empirical testing, the extended TAM repositions the classical cognitive constructs—perceived usefulness and perceived ease of use—within a broader institutional environment characterised by trust, legitimacy, transparency, and community support. These institutional dimensions operate as contextual conditions that shape how technological attributes are interpreted, evaluated, and translated into behavioural intention, particularly in governance-intensive and policy-relevant settings.

Within this consolidated framework, two analytically distinct yet interdependent dimensions can be identified. The behavioural dimension captures individual-level cognitive assessments that have traditionally formed the core of technology acceptance research, while the institutional dimension reflects collective-level conditions linked to governance arrangements, institutional credibility, and social endorsement. By explicitly linking cognitive evaluations to institutional context, the extended TAM conceptualises technology acceptance as a socially embedded process, in which individual judgements about usefulness or usability are inseparable from broader perceptions of institutional reliability, normative alignment, and procedural fairness.

The framework proposed here does not claim predictive validity or empirical generalisation. Its contribution lies in offering a conceptually integrated lens capable of organising dispersed insights from behavioural psychology, technology acceptance research, and governance studies into a coherent analytical structure. This consolidation provides a structured point of departure for future empirical work and prepares the ground for the interpretative discussion that follows.

## 4 Discussion

This section examines the implications of the findings derived from the bibliometric mapping, structured expert elicitation, and conceptual framework developed in the preceding sections. Rather than treating these findings as empirical validation of causal relationships, the discussion situates them within broader debates in environmental psychology, behavioural public policy, and sustainability transitions. The analysis focuses on how behavioural and institutional determinants jointly shape citizens’ acceptance of green technologies, on the contribution of extending the Technology Acceptance Model (TAM) to institutionally complex contexts, and on the theoretical and policy-relevant insights that emerge from this integrative framework.

### 4.1 Integration of behavioural and institutional determinants

The findings of this study indicate that behavioural intention and perceived usefulness, core constructs of the original Technology Acceptance Model (TAM), are insufficient to fully account for the acceptance of sustainable technologies when considered independently of their institutional and normative context. Evidence from both the Analytic Hierarchy Process (AHP) and the bibliometric co-occurrence analysis points to the central role of institutional variables—particularly trust, legitimacy, and transparency—in structuring contemporary understandings of green technology adoption. This pattern is consistent with comparative research showing that institutional quality and environmental governance arrangements shape sustainability-related outcomes across diverse socio-political settings (Hussain and Dogan, 2021).

Within the extended TAM proposed in this study, institutional determinants do not displace individual cognitive evaluations. Instead, they condition the environment in which such evaluations acquire behavioural significance. Trust and legitimacy operate as contextual anchors, reinforcing perceptions of credibility, fairness, and reliability in both technological solutions and the institutions that promote them. This interpretation aligns with research in environmental psychology emphasising the role of institutional credibility and procedural justice in motivating pro-environmental behaviour (Ajina et al., 2024), while governance-focused studies show that transparency contributes to acceptance by reducing uncertainty and signalling accountability in public decision-making processes (Jannah et al., 2025).

From an integrative standpoint, the extended TAM captures a dual structure of technology acceptance. Behavioural determinants rooted in individual cognition—such as perceived usefulness and perceived ease of use—remain important drivers of adoption, but institutional determinants operate at a collective level by shaping the interpretive frame through which individuals evaluate technological innovations. As a result, acceptance emerges as a socially embedded process, less dependent on isolated rational choice and more closely tied to perceptions of institutional legitimacy, transparency, and trustworthiness in the context of sustainability transitions.

### 4.2 Conceptual convergence and robustness of the extended TAM

The robustness of the extended Technology Acceptance Model can be assessed by examining convergence patterns observed across bibliometric co-occurrence structures and the analytical synthesis developed in this study. Rather than serving as statistical validation, this analysis evaluates whether the proposed integration of behavioural and institutional dimensions aligns with the dominant ways in which green technology adoption is framed in the contemporary literature.

**Figure 6:**
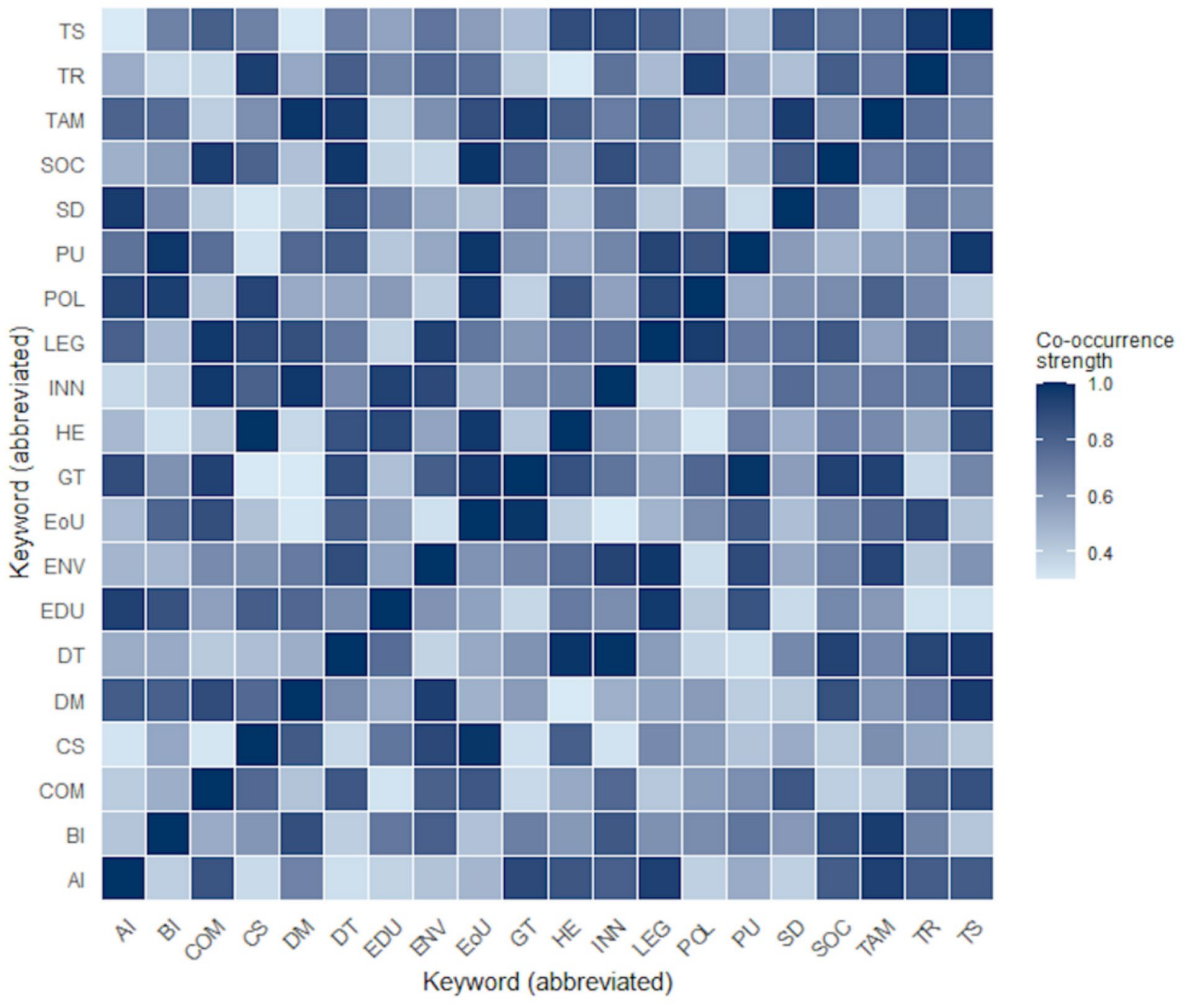
Heatmap of the 20 most frequent co-occurring author keywords in the 2021–2025 Scopus corpus. Source: authors’ elaboration based on Scopus data (retrieved October 2025).

As illustrated in Figure 6, the heatmap derived from the most frequently used author keywords in the 2021–2025 Scopus corpus highlights recurrent associations between classical TAM constructs, such as perceived usefulness, perceived ease of use, and behavioural intention, and institutional concepts including trust, legitimacy, transparency, and community support. These associations indicate that technology acceptance in sustainability-oriented contexts is rarely conceptualised as a purely individual or cognitive process, but is instead situated within broader institutional and normative frameworks.

This pattern is further supported by the quantitative descriptors reported in Table 3, which summarises keyword frequencies and total link strength values for the 20 most interconnected concepts in the corpus. Institutional constructs—most notably trust and legitimacy—display both high frequency and strong interconnectedness, comparable to or exceeding those of traditional behavioural variables. These indicators should not be interpreted as evidence of causal influence or predictive hierarchy, but as signals of the central and integrative position that institutional dimensions occupy within contemporary research on sustainable technology adoption.

**Table 3:** Top 20 author keywords by frequency and total link strength in the 2021–2025 Scopus corpus.

Keyword	Frequency	Total link strength	Cluster/thematic domain
Trust	58	249.2	Institutional confidence
Technology acceptance model (TAM)	88	244.8	Behavioural theory
Perceived usefulness	52	236.7	Cognitive beliefs
Ease of use	47	225.3	Cognitive beliefs
Legitimacy	42	214.6	Institutional perception
Transparency	39	202.9	Governance and ethics
Community support	35	197.4	Social endorsement
Behavioural intention	31	186.2	Behavioural outcome
Sustainability	43	175.9	Environmental orientation
Green technology	41	170.4	Environmental innovation
Artificial intelligence	30	158.7	Technological application
Higher education	31	154.2	Educational context
Digital transformation	28	149.5	Technological integration
Policy	26	144.8	Governance dimension
Environmental behaviour	29	142.1	Sustainability psychology
Decision-making	23	138.3	Cognitive process
Citizen science	22	133.8	Participatory engagement
Social norms	21	128.4	Societal framework
Innovation	20	126.1	Technological change
Education	25	124.9	Learning and dissemination

Source: authors’ elaboration based on Scopus data (retrieved October 2025).

The convergence between behavioural and institutional constructs observed in Figure 6 and Table 3 is consistent with a growing body of literature emphasising the role of institutional quality, governance arrangements, and social legitimacy in shaping sustainability-related outcomes (Hussain and Dogan, 2021). Research in behavioural public policy similarly indicates that citizens’ responses to technological and policy interventions are mediated by perceptions of transparency, fairness, and procedural legitimacy rather than by instrumental considerations alone (Fleiß et al., 2024). At a conceptual level, these convergence patterns reinforce the analytical rationale underpinning the extended TAM proposed in this study, suggesting that the integration of institutional dimensions systematises an interpretative orientation already implicit across diverse strands of research (Alessandro et al., 2021).

### 4.3 Policy implications for behavioural and institutional design

The integration of institutional dimensions into behavioural models provides a robust conceptual foundation for the design of policies aimed at promoting sustainable technology adoption. The analysis indicates that citizens’ willingness to engage with green technologies is influenced not only by perceptions of efficiency or usability, but also by how institutional actors are perceived in terms of credibility, transparency, and procedural fairness. For policymakers and public organisations, this suggests that technological solutions alone are unlikely to achieve broad acceptance unless they are accompanied by sustained efforts to strengthen institutional trust and legitimacy.

Empirical research on sustainability-oriented policy interventions supports this interpretation, showing that environmental knowledge, regulatory frameworks, and institutional signals interact with attitudes and perceptions to shape sustainable consumption patterns (Li et al., 2025). In practice, policy initiatives may therefore benefit from prioritising transparent communication of environmental objectives, explicit accountability mechanisms, and participatory processes that enable citizens to engage meaningfully with decision-making. These approaches are consistent with the principles of behavioural public policy, which emphasise that perceptions of legitimacy and fairness shape how individuals interpret policy signals and respond to public interventions (Fleiß et al., 2024).

These implications should be interpreted as guiding orientations rather than prescriptive solutions. The extended TAM framework does not advocate specific instruments or intervention packages; instead, it highlights institutional context as a critical lens through which the effectiveness of sustainability policies can be evaluated, adapted, and improved over time.

### 4.4 Limitations and directions for future research

Despite its integrative scope, this study is subject to several limitations. First, the bibliometric corpus was restricted to English-language journal articles indexed in Scopus, which may underrepresent non-Western research traditions, locally grounded studies, and forms of grey literature central to community-based sustainability initiatives. Consequently, the conceptual patterns identified here reflect dominant strands of international academic discourse rather than a comprehensive representation of global perspectives on green technology adoption.

Second, the Analytic Hierarchy Process was employed as a structured expert elicitation and organisational tool rather than as a method of empirical validation. Although this approach facilitated conceptual clarification and comparative reflection across behavioural and institutional dimensions, it cannot substitute for analyses based on primary behavioural data. Future research could operationalise the extended TAM through large-scale survey designs, experimental studies, or mixed-method approaches, including structural equation modelling, to examine how institutional and behavioural determinants interact across specific technological and policy contexts.

Overall, the extended Technology Acceptance Model proposed in this study should be viewed as a flexible conceptual scaffold rather than a closed explanatory system. Its primary contribution lies in structuring future inquiry into how cognitive evaluations, institutional conditions, and social contexts jointly shape the adoption of green technologies, while providing a coherent basis for empirical extension and policy-relevant application.

## 5 Conclusion

This study integrates behavioural, institutional, and social perspectives on technology acceptance within a coherent conceptual framework that is analytically grounded and relevant for policy-oriented inquiry. By developing a conceptually extended version of the Technology Acceptance Model (TAM), the article advances an integrative understanding of citizens’ acceptance of sustainable technologies that goes beyond individual cognitive evaluations. Drawing on bibliometric mapping, semantic analysis, and structured expert elicitation through the Analytic Hierarchy Process (AHP), the analysis highlights the central role of institutional conditions, specifically trust, legitimacy, transparency, and community support, in shaping how technological attributes are interpreted and translated into behavioural intention.

The findings indicate that technology adoption in environmental contexts cannot be adequately captured as a purely individual or instrumental process. Instead, acceptance emerges within a broader social and institutional setting in which perceptions of credibility, fairness, and procedural transparency condition whether perceived usefulness and ease of use effectively lead to adoption. In this sense, the study contributes to the theoretical evolution of TAM by situating cognitive evaluations within governance and institutional contexts that shape behavioural responses, particularly in sustainability-oriented and public-sector settings.

From a practical perspective, the framework developed here offers insights relevant for sustainability governance and public innovation. Rather than focusing exclusively on technological performance or economic incentives, the analysis underscores the importance of institutional credibility and participatory legitimacy as enabling conditions for successful ecological transitions, especially in urban and public policy contexts where citizen trust and perceptions of procedural fairness often shape the reception and effectiveness of green initiatives (Wamsler et al., 2024).

Looking ahead, the extended TAM proposed in this study should be regarded as an open conceptual framework that invites further empirical exploration. Future research may strengthen its analytical and policy relevance through quantitative validation, longitudinal designs, and the use of reproducible open-data sources that capture behavioural dynamics over time and across contexts. Overall, the analysis reinforces a central insight: sustainable transformation depends not only on the availability or performance of green technologies, but also on the institutional environments in which they are introduced and governed. Trust, in this sense, is not an auxiliary consideration but a foundational condition underpinning citizens’ willingness to engage with sustainable innovation.

## Data Availability

The original contributions presented in the study are included in the article/Supplementary material, further inquiries can be directed to the corresponding author.
