# Supplementary material for: Behavioural and institutional drivers of green technology adoption: an extended technology acceptance model for sustainability transitions
Source: Front Psychol. 2026 Mar 13;17:1723942. doi: 10.3389/fpsyg.2026.1723942 (PMC13021414; doi:10.3389/fpsyg.2026.1723942)
Supplement: Supplementary file 2 [file Table_1.docx]

Supplementary Material

# Supplementary Text 1. Scopus search query used for data collection

The bibliometric dataset described in Section 2.2 was obtained from the Scopus database using the following Boolean query:

(TITLE-ABS-KEY(technology acceptance model OR TAM OR adoption OR acceptance OR "intention to use"))
AND (TITLE-ABS-KEY(psychological* OR behavior* OR cognitive* OR perception OR trust))
AND (TITLE-ABS-KEY(social* OR community* OR cultural* OR societal OR "social norms"))
AND (TITLE-ABS-KEY(environment* OR sustainability OR "green technology" OR "environmental innovation"))
AND PUBYEAR > 2020 AND PUBYEAR < 2026
AND DOCTYPE(ar)
AND ( LIMIT-TO ( SUBJAREA,"SOCI" )
 OR LIMIT-TO ( SUBJAREA,"PSYC" )
 OR LIMIT-TO ( SUBJAREA,"ENVI" )
 OR LIMIT-TO ( SUBJAREA,"BUSI" )
 OR LIMIT-TO ( SUBJAREA,"DECI" )
 OR LIMIT-TO ( SUBJAREA,"COMP" ) )

The search retrieved 789 peer-reviewed journal articles and was conducted in October 2025. All bibliographic metadata were exported in .csv format for subsequent bibliometric and semantic analyses. Wildcard operators were used to capture spelling variants and conceptually related terms across disciplinary domains.

# Supplementary Data

Supplementary material is not typeset so please ensure that all information is clearly presented, the appropriate caption is included in the file and not in the manuscript, and that the style conforms to the rest of the article.

# Supplementary Figures and Tables

All figures were generated using R (version 4.3.2) with the ggplot2 package. Each figure is exported in TIFF format (300 dpi) with white background and colours harmonised with the article’s visual scheme.

## Supplementary Figures


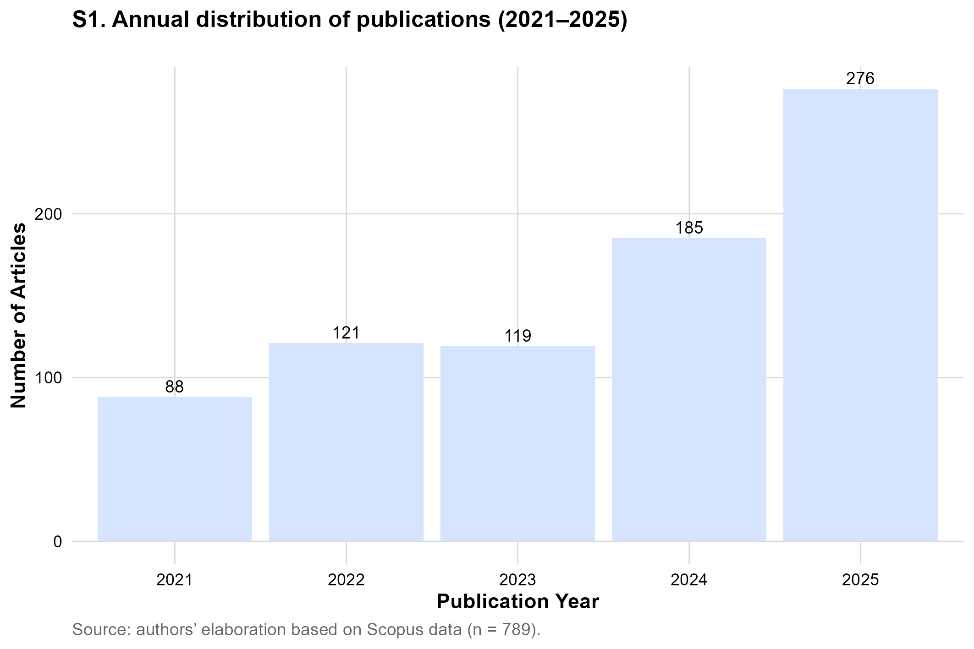


**Figure S1.** Annual distribution of publications indexed in Scopus between 2021 and 2025. The steady increase after 2021 reflects the growing academic interest in the intersection between technology acceptance, behavioural psychology, and environmental sustainability. *Source: authors’ elaboration based on Scopus data (n = 789).*


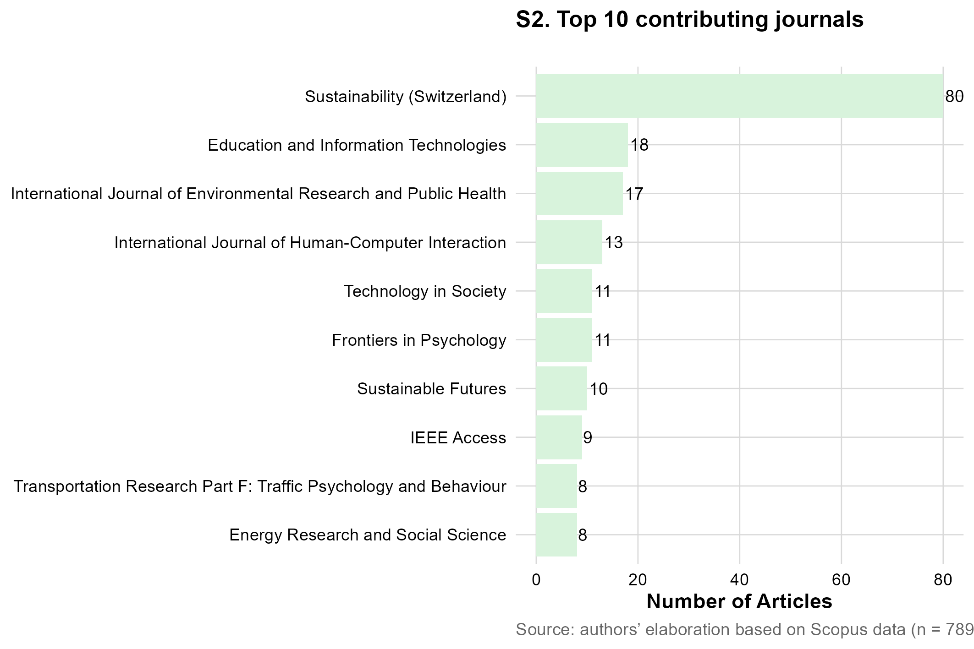


**Figure S2.** Top ten academic journals publishing research related to technology acceptance, environmental psychology, and sustainability behaviour. Journals from psychology, information systems, and environmental sciences dominate the corpus. *Source: authors’ elaboration based on Scopus data (n = 789).*


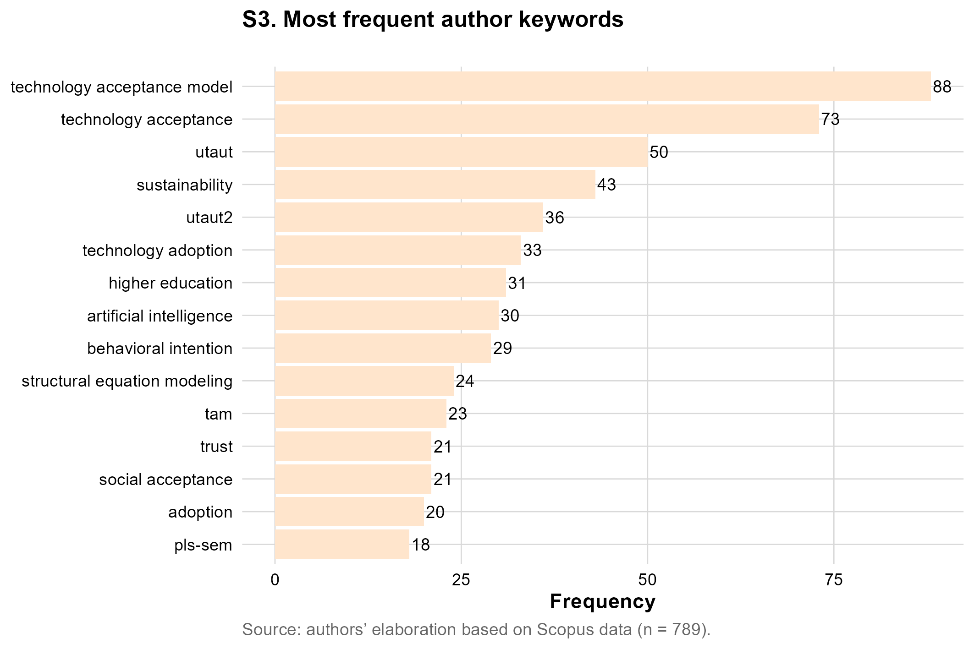


**Figure S3.** Frequency of author-supplied keywords in the analysed corpus. Concepts such as “technology acceptance model”, “trust”, and “sustainability” form the central thematic clusters connecting behavioural and environmental research. *Source: authors’ elaboration based on Scopus data (n = 789).*


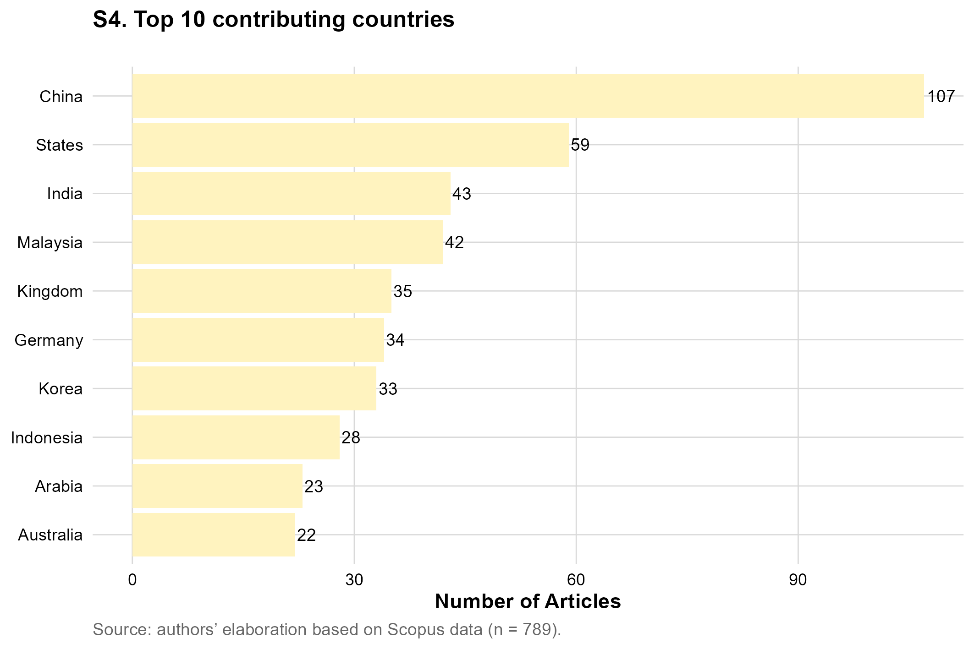


**Figure S4.** Distribution of publications by country based on author affiliations. Research activity is concentrated in Europe and East Asia, with growing contributions from Latin America. *Source: authors’ elaboration based on Scopus data (n = 789).*


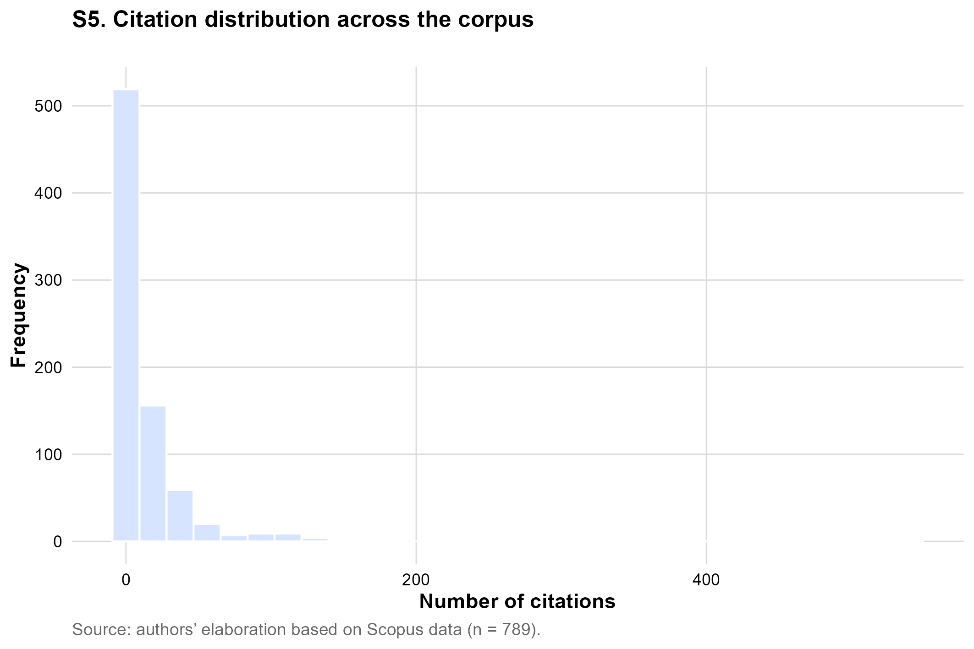


**Figure S5.** Citation distribution of the 789 analysed articles. Most studies receive between 5 and 20 citations, indicating a rapidly emerging field still in consolidation. *Source: authors’ elaboration based on Scopus data (n = 789).*
